# Incidence of and factors associated with catheter-related bloodstream infection in patients with advanced solid tumors on home parenteral nutrition managed using a standardized catheter care protocol

**DOI:** 10.1186/s12879-017-2469-7

**Published:** 2017-05-30

**Authors:** Pankaj G. Vashi, Natasha Virginkar, Brenten Popiel, Persis Edwin, Digant Gupta

**Affiliations:** Cancer Treatment Centers of America® (CTCA) at Midwestern Regional Medical Center, 2520 Elisha Ave, Zion, Illinois 60099 USA

**Keywords:** Bloodstream infections, Cancer, Home parenteral nutrition, Standardized protocol

## Abstract

**Background:**

Catheter-related bloodstream infections (CRBSIs) are associated with high morbidity and mortality as well as increased medical costs. Cancer patients, who are often immunocompromised, are susceptible to CRBSI while receiving home parenteral nutrition (HPN). We evaluated the incidence of and factors associated with CRBSIs in cancer patients undergoing HPN managed using a standardized catheter care protocol.

**Methods:**

This is a retrospective cohort study of 335 cancer patients receiving HPN between January 2012 and July 2015. The primary outcome of interest was the incidence of CRBSI expressed as events per 1000 HPN days. HPN days were calculated from the start date with the home infusion provider until the discontinuation of HPN, or the removal of the venous access device (VAD), or the death of the patient. The VADs used were either peripherally inserted central catheters (PICCs) or a subcutaneous implanted port or tunneled central catheters (TCCs). Univariate Poisson regression analyses were used to determine the variables associated with CRBSIs.

**Results:**

Of 335 patients, 193 were females and 142 were males. The most common cancer types were colorectal, pancreatic, ovarian and stomach. A total of 408 VADs in 335 patients were studied, covering a total of 29,403 HPN days. Of 408 VADs, 206 (50.5%) were ports, 191 (46.8%) were PICCs, and 7 (2.7%) were TCCs. The median duration of HPN was 54 days. A total of 16 CRBSI episodes were recorded (8 in ports, 7 in PICCs and 1 in TCCs). The median duration from the start of HPN to the development of CRBSI episodes was 43.5 days. The overall incidence of CRBSI per 1000 HPN days was 0.54 (95% confidence interval: 0.32–0.86). Upon univariate analysis, no variables were found to be statistically significantly associated with CRBSI incidence.

**Conclusions:**

We found a low rate of CRBSI following a standardized catheter maintenance protocol in a high-risk oncology population undergoing HPN.

## Background

Cancer patients often require central venous access for continuous infusion of chemotherapeutic agents, hydration, and parenteral nutrition (PN) [1]. Other indications for the placement of these catheters in patients with cancer include poor peripheral access, frequent need to administer blood products or antibiotics or to draw blood, a prolonged treatment course and use of vesicant drugs [2].

Earlier, long-term venous access devices (VADs) were described by Broviac et al. and then by Hickman et al. [3, 4]. Central venous devices are either simple catheters, such as tunneled Hickman catheters, or catheters linked to a subcutaneous port. Both types of VADs, simple or port catheter, are placed percutaneously via a subclavian route or a jugular route under sonographic guidance [5]. Implanted subcutaneous ports or tunneled catheters are placed in the operating room or a designated minor procedure room in order to adhere to an aseptic environment and strict sterile technique. Intraoperative fluoroscopy is often used to guide the catheter into the correct position. Peripherally inserted central catheters (PICCs), on the other hand, are usually placed ultrasound guided in the intervention radiology department using sterile precautions.

Although the use of long-term VADs has enhanced the lives of oncology patients, it has added a new set of challenges [6]. While totally implantable access ports have the advantages of not requiring an external dressing, allowing more patient activity and requiring only monthly maintenance flushing [7, 8], these devices still have a reported complication rate of 11% to 25% [7, 9, 10]. Complications of catheterization include those associated with VAD insertion such as pneumothorax and arterial and nerve injuries as well as those associated with long-term VAD use such as thrombosis and catheter-related bloodstream infection (CRBSI) [11–14]. Other complications include embolization of a ruptured distal part of the catheter, migration of the tip of the catheter, fibrin-sheath obstruction of the catheter, embolization of a guidewire during placement of the central venous device and leakages [5, 15].

Cancer patients, who are often immunocompromised, are more susceptible to CRBSI while receiving total parenteral nutrition [16]. The major causes of CRBSI are migration of the skin organisms at the insertion site into the catheter tract and contamination of the catheter hub [17]. This incidence of CRBSI ranges from 0.05 to 6.8 infections per 1000 catheter days [17, 18]. Success rates of 60% to 91% have been reported using antimicrobial therapy to treat CRBSIs, although often the device has to be removed [19]. The organisms most commonly cultured are gram-positive (70%; Coagulase negative Staphylococci, *Staphylococcus aureus* and Enterococci], gram-negative (15%; *Escherichia coli*), fungal (8%; Candida), and anaerobic bacteria (7%) [20].

Few observational studies (with none conducted in the US) have quantified the incidence of CRBSI in patients with solid tumors receiving active oncologic treatment while undergoing home parenteral nutrition (HPN) [18, 21–25]. The aims of the present study were to evaluate the following in a case series of patients with advanced solid tumors treated at a tertiary care cancer center in the US: 1) The incidence of CRBSI while receiving HPN using a standardized catheter care protocol; 2) Factors associated with CRBSI development.

## Methods

### Study design and patient population

This is a retrospective cohort study of a consecutive case series of patients with solid tumors receiving HPN between January 2012 and July 2015 while being treated at Cancer Treatment Centers of America^®^ (CTCA) at Midwestern Regional Medical Center. We included a consecutive case series of patients to avoid non-response and minimize the probability of selection bias. The criteria for receiving HPN were based on the American Society for Parenteral and Enteral Nutrition (ASPEN) guidelines [26] and included the following: (1) Patients receiving active anticancer treatment who are malnourished and who are anticipated to be unable to ingest and/or absorb adequate nutrients for a prolonged period of time; (2) Eastern Cooperative Oncology Group (ECOG) performance status score of <= 2 (minimum score of 0 indicates a normal person with no limitations, whereas a maximum score of 5 indicates a dead person); (3) Evaluation and approval by a multi-disciplinary nutrition and metabolic support team (NMST) comprised of a physician, dietitian, nurse, pharmacist and care manager.

The present study was conducted according to the guidelines laid down in the Declaration of Helsinki and was approved by the Institutional Review Board (IRB) at CTCA Midwestern Regional Medical Center. The need for informed consent was waived by the IRB because there was no direct patient contact in this study. This study involved collection of existing data from patient records in such a manner that subjects cannot be identified, directly or through identifiers linked to the subjects. Patient records/information was anonymized and de-identified prior to analysis.

### Venous access devices

The VADs used in our patients were either PICCs, or a subcutaneous implanted port or tunneled central catheters (TCCs). The choice of the type of VAD used in each patient was primarily based on the provider’s preference. The ports were inserted by a surgical oncologist. All ports were inserted in the upper chest through a subclavian vein approach. The placement was confirmed by an X-ray before using the port. The PICC lines were inserted under ultrasound guidance into the brachial or basilic veins by a trained nurse or an interventional radiologist. The TCCs were inserted by a surgeon. All VADs were inserted with maximal barrier precautions and skin antisepsis with 2% chlorhexidine swabs as part of a pre-packaged kit. The barrier precautions used for VADs depended upon the type of access. All the ports were inserted in the sterile operation room by the surgeon. After washing the hands with soap and water with scrubbing, the surgeon wore a sterile gown and gloves for the procedure like any major surgery. The PICC and TCC lines were inserted in the designated interventional radiology room by the interventional radiologist who also followed the same routine as the surgeons for sterile barrier. Sterile solution (2% chlorhexidine) was used for all the patients as a barrier with sterile draping covering up the insertion site. The appropriate central position of the tip of the catheter was consistently verified, either by fluoroscopy during the procedure or by chest X-ray after the procedure.

### Standardized VAD care protocol

All patients received HPN from a single home infusion company. Patients were managed by 12 different branches of the same home infusion company across the country. Patients received detailed pre-discharge teaching by a team of hospital and home infusion nurses, registered dietitians, and a dedicated case manager prior to going home with PN. Every patient was evaluated for the presence of a suitable environment at home for HPN which included clean running water, designated area in the refrigerator to store the PN bags, access of communication (cell phone or a land line), clean designated area for preparation of the PN bag, and good family support system that understands the steps for sterile infusion and basic monitoring devices like a weighing machine, thermometer to check temperature and blood sugar monitoring device in selected patients. Every patient had to have a responsible caregiver at home which was defined as someone who understood step-by-step PN preparation process, troubleshooting and identifying any potential complications.

Written as well as visual instructions on step-by-step process of caring for the VAD were given to the patient and the caregiver before their transition to HPN. A home health nurse for each patient was arranged by the same infusion company. Patients in the study were managed using a specific catheter care protocol that included: a strict aseptic flushing and dressing change procedure; weekly sterile dressing changes with use of Chloraprep®; and the application of MicroClave® connectors and SwabCaps® on all lumens that were not in use. A weekly assessment was performed that provided details about the patients’ clinical status, compliance with catheter care and HPN, and catheter status. Complete blood count and serum chemistry were conducted weekly and critical values were reported to the metabolic support team immediately. Printed materials and custom multimedia videos were used to reinforce infusion technique and patient/caregiver compliance with catheter maintenance protocol. A member of the NMST was available to answer any questions regarding these videos.

### CRBSI diagnosis and management

The diagnosis and management of CRBSI were conducted according to the clinical practice guidelines provided by the Infectious Diseases Society of America (IDSA) [27]. Any patient who developed unexplained high grade fever (101 degrees F or higher) was evaluated for CRBSI by performing blood cultures. The blood cultures (both peripheral and central) were performed in the local emergency room. In the case of a positive blood culture, antibiotics were selected according to the sensitivity of the responsible microorganism(s). Antibiotic administration and catheter removal were conducted according to the IDSA guidelines [27].

### Statistical analysis

The *primary outcome* of interest was the incidence of CRBSI. Incidence rates of CRBSI were expressed as events per 1000 HPN days. This is the ratio of the number of VAD-related episodes of infection to the total number of HPN days during the study period multiplied by 1000. Follow-up continued until either the VAD was removed or HPN stopped. Loss to follow-up was considered when contact with the patient was lost while still receiving HPN. HPN days were calculated from the start date with the home infusion provider until the discontinuation of HPN, or the removal of the VAD, or the death of the patient. The *primary independent variable* of interest was the type of VAD. The following variables were evaluated as potential *covariates* or *confounders* because of their potential to be associated with the key outcome of interest (incidence of CRBSI): age, gender, tumor type, Subjective Global Assessment (SGA), ECOG performance status, stage at diagnosis, body mass index (BMI) and serum albumin. The SGA is a clinical technique that combines data from subjective and objective aspects of medical history (weight change, dietary intake change, gastrointestinal symptoms, and changes in functional capacity) and physical examination (loss of subcutaneous fat, muscle wasting, ankle or sacral edema, and ascites). After evaluation, patients were categorized into 3 distinct classes of nutritional status; well nourished (SGA-A), moderately malnourished (SGA-B) and severely malnourished (SGA-C) as described by Detsky et al. [28]. The distribution of these potential confounders across the different types of VADs was examined using the computation of standardized mean differences in accordance with the guidance provided by Austin 2011 [29]. A standard difference of less than 0.2 was taken to indicate a negligible difference in the mean or prevalence of a covariate between VAD types. The duration of VADs was compared across the different types of VADs using the non-parametric Kruskal-Wallis test with pair-wise comparisons. CRBSI incidence rates across the VAD types were compared using the Fisher and Mid-P exact tests adjusted for HPN-days (using the number of HPN days as the denominator).

Univariate Poisson regression was used to determine which variables were associated with CRBSIs. Since the amount of exposure to the risk of developing CRBSI was different across patients, the “natural log of HPN days” was added as an “offset” variable in all Poisson regression analyses. The Pearson dispersion statistic was used to evaluate the presence of overdispersion in the models. Values of Pearson dispersion statistic greater than 1 were taken to indicate overdispersion. In such a scenario, scaling of standard errors and robust (or sandwich) variance estimators were employed, to adjust for overdispersion in the models. When using scaling or robust variance estimators, the parameter estimates are the same as in the previous model fit, but the standard errors are increased, resulting in more conservative statistical tests. Scaling or applying a robust variance estimate allows the fit of the model to better reflect the true underlying distribution. The effect of individual variables on the incidence of CRBSI was expressed as incidence rate ratios (IRRs) calculated by exponentiating the estimated parameters of the Poisson models. The corresponding 95% confidence intervals (CIs) and *p*-values were also reported.

No formal sample size calculations were conducted for this analysis. All data were analyzed using IBM SPSS version 23.0 (IBM, Armonk, NY, USA). All analyses were two-tailed, and a difference was considered to be statistically significant if the *p* value was <= 0.05.

## Results

### Patient characteristics

The final study sample consisted of 335 consecutive patients of which 193 were females (57.6%) and 142 were males (42.4%) with an overall mean age of 53.7 years. All eligible patients during the study period were included with no exclusions at any stage. There were no losses to follow-up and all patients were included in the final analysis. The most common cancer types were colorectal, pancreatic, ovarian, and stomach. The majority of our patient population was moderately to severely malnourished (81.5%) at baseline and had received chemotherapy or radiotherapy during HPN (73.1%). The median duration of HPN was 54 days. Tables 1 and 2 describe the baseline characteristics of our patient cohort in greater detail.

**Table 1:** Baseline patient characteristics (Categorical Variables); *N* = 335

Characteristic	Categories	Number (Percent)
Gender	Male	142 (42.4)
Cancer site	Colorectal	71 (21.2)
	Pancreas	59 (17.6)
	Ovary	35 (10.4)
	Stomach	29 (8.7)
	Cervix	17 (5.1)
	Esophagus	16 (4.8)
	Lung	15 (4.5)
	Others	93 (27.8)
Cancer stage at diagnosis	Stage 0	1 (0.3)
	Stage I	26 (7.8)
	Stage II	53 (15.8)
	Stage III	98 (29.3)
	Stage IV	157 (46.9)
Chemotherapy during HPN	Yes	208 (62.1)
Radiotherapy during HPN	Yes	73 (21.8)
SGA at HPN Start	Well-nourished	62 (18.5)
	Moderately Malnourished	175 (52.2)
	Severely Malnourished	98 (29.3)
ECOG performance status	0	11 (3.3)
	1	86 (25.7)
	2	132 (39.4)
	3	101 (30.1)
	4–5	5 (1.5)

*ECOG* Eastern Cooperative Oncology Group, *HPN* home parenteral nutrition, *SGA* Subjective Global Assessment

**Table 2:** Baseline patient characteristics (Continuous Variables); *N* = 335

Characteristic	Mean (SD)	Median	Range
Age (years) at start of HPN	53.7 (9.3)	55.2	22.8–84.5
Weight (kg) at start of HPN	69.2 (18.2)	67.7	31–145.4
BMI (kg/m^2^) at start of HPN	24.2 (6.2)	23.1	12.2–57.1
Albumin (g/dl) at start of HPN	2.7 (0.64)	2.7	0.9–5.9
HPN Duration (days)	87.7 (108.4)	54	0–972

*BMI* body mass index, *g/dl* grams per deciliter, *HPN* home parenteral nutrition, *kg* kilograms, *kg/m* ^*2*^ kilograms per meter squared, *SD* standard deviation

The primary indications for using HPN in these patients were poor oral intake in spite of aggressive nutrition counseling by a dietitian, failure of oral supplementations and appetite stimulants (*n* = 123; 36.7%), weight loss/malnutrition (*n* = 84; 25.1%), bowel obstruction (*n* = 42; 12.5%) and post cytoreductive surgery and hyperthermic intraperitoneal chemotherapy (*n* = 33; 9.9%). All patients were followed up until either the VAD was removed or HPN stopped. HPN was discontinued in most patients on recovery of oral nutrition (*n* = 120; 35.8%), worsening of clinical state (*n* = 85; 25.4%), death (*n* = 77; 23%) and transfer to other facility (*n* = 33; 9.9%); the reasons for HPN discontinuation were not statistically significantly different across the 3 VAD types.

### VADs

All the VADs placed in this study were used for HPN. In those patients who were receiving HPN and chemotherapy, the VAD was used for both but never at the same time. Among the 335 unique patients described above, the study included 408 VADs covering a total of 29,403 HPN days (median 46 days). Of the 408 VADs, 206 (50.5%) were ports, 191 (46.8%) were PICCs, and 11 (2.7%) were TCCs. Ports and PICCs were the only two VADs offered at our institution. The 11 TCCs were placed at a different institution before those patients visited us. Table 3 displays the characteristics of VADs in greater detail (this table is based on the total number of VADs and not the total number of unique patients). The median duration of HPN for ports was significantly greater than the median HPN duration for PICCs (53 days versus 41 days; *p* = 0.02). Table 4 displays the standardized mean differences in the distribution of baseline characteristics across the 3 VAD types. A standardized mean difference of greater than equal to 0.20 in a particular baseline characteristic was considered to indicate an imbalance between the 2 VAD groups. As can be seen from Table 4, the Port and PICC groups were different from each other with regard to BMI; the PICC and TCC groups were different from each other with regard to age, gender, cancer stage and ECOG performance status; the Port and TCC groups were different from each other with regard to age, BMI, gender, cancer stage and ECOG performance status.

**Table 3:** Characteristics of and Infections Associated with VADs; *N* = 408

	Port	PICC	TCC	Total
Characteristics				
Number (%) of VADs	206 (50.5)	191 (46.8)	11 (2.7)	408 (100)
Median HPN Days#	53	41	88	46
Total HPN Days	16,929	11,401	1073	29,403
Mean Age (years)	53.6	53.9	58.6	53.8
Mean BMI (kg/m^2^)	23.5	25.1	25.3	24.3
Mean Serum Albumin (g/dl)	2.8	2.8	2.9	2.8
Gender, % Male	43.7	43.5	54.5	43.9
Cancer Stage at Diagnosis, % Stage IV	51.5	43.5	27.3	47.1
SGA at HPN Start, % well-nourished	23.3	19.9	18.2	21.6
ECOG Performance Status, % 0–2	67	71.7	81.8	69.6
Infection				
Number of CRBSI	8	7	1	16
Number of CRBSI/1000 HPN Days (95% CI)*	0.47 (0.20–0.93)	0.61 (0.25–1.3)	0.93 (0.02–5.2)	0.54 (0.32–0.86)
Days to CRBSI, Median (Range)	83.5 (13–420)	30 (0–95)	187 (187–187)	43.5 (0–420)

*CI* confidence interval, *CRBSI* catheter-related bloodstream infection, *ECOG* Eastern Cooperative Oncology Group, *HPN* home parenteral nutrition, *PICC* peripherally inserted central catheter, *SGA* Subjective Global Assessment, *TCC* tunneled central catheter, *VAD* venous access device

#Port versus PICC *p* = 0.02

Port versus TCC *p* = 0.99

PICC versus TCC *p* = 0.34

*Port versus PICC *p* = 0.61

Port versus TCC *p* = 0.52

PICC versus TCC *p* = 0.65

**Table 4:** Standardized Mean Differences in Baseline Characteristics across VADs; *N* = 408

Characteristics	Port vs. PICC	PICC vs. TCC	Port vs. TCC
Mean Age (years)	0.03	0.52*	0.58*
Mean BMI (kg/m^2^)	0.27*	0.04	0.35*
Mean Serum Albumin (g/dl)	0.00	0.16	0.16
Gender, % Male	0.00	0.22*	0.22*
Cancer Stage at Diagnosis, % Stage IV	0.16	0.34*	0.51*
SGA at HPN Start, % well-nourished	0.08	0.04	0.13
ECOG Performance Status, % 0–2	0.10	0.24*	0.34*

*BMI* body mass index, *ECOG* Eastern Cooperative Oncology Group, *g/dl* grams per deciliter, *HPN* home parenteral nutrition, *kg/m2* kilograms per meter squared, *PICC* peripherally inserted central catheter, *SGA* Subjective Global Assessment, *TCC* tunneled central catheter, *VAD* venous access device

*Values >0.20

### CRBSIs

#### Incidence

Of 335 patients, 15 (4.5%) patients experienced CRBSI. One patient experienced 2 episodes of CRBSI. As a result, for a total of 408 VADs, the number of CRBSI cases was 16 (3.9%). Table 3 displays the overall incidence of CRBSI as well as incidence stratified by the 3 VAD types. The overall incidence of CRBSI per 1000 HPN days was 0.54 (95% CI: 0.32 to 0.86). Of the 16 CRBSI cases, 8 occurred in ports, 7 in PICCs and 1 in TCCs. There were no statistically significant differences in the incidence rates of CRBSI across the 3 types of VADs. The median duration from the start of HPN to the development of CRBSI episodes was 43.5 days.

The majority of cases with CRBSIs were either moderately malnourished (*n* = 6) or severely malnourished (*n* = 6) at the start of HPN therapy. Eight cases occurred in males and 8 in females. Ten (62.5%) cases occurred in patients with stage 4 disease. Fourteen (87.5%) cases received chemotherapy during HPN while only 3 (18.8%) received radiotherapy. In 15 out of 16 cases of CRBSI, the VAD was removed because of infection. Similarly, in 15 out of 16 cases, patients were hospitalized because of infection. The case in whom the VAD was not removed was not the one who was not hospitalized. With respect to microbiology, CRBSIs were caused by *Staphylococcus epidermidis* (6 cases), candida (3 cases), Coagulase-negative Staphylococci (3 cases), Methicillin-resistant *Staphylococcus aureus* (1 case), *Klebsiella pneumoniae* (1 case), *Escherichia coli* (1 case) and Enterococcus faecium (1 case).

#### Factors associated with CRBSI

Table 5 displays the results of univariate Poisson regression analyses evaluating the factors associated with CRBSI. No variables were found to be statistically significantly associated with CRBSI incidence. Type of VAD had no effect on the incidence of CRBSI. Severely malnourished patients and those with metastatic disease had almost twice the risk of CRBSI compared to well-nourished and non-metastatic patients respectively; however the results did not attain statistical significance. Similarly, serum albumin <3.5 g/dl was associated with almost thrice the risk of CRBSI compared to serum albumin > = 3.5 g/dl, however, the finding was not statistically significant. No multivariate Poisson regression analyses were conducted.

**Table 5:** Univariate Poisson Regression Analysis of the Factors Associated with CRBSI; *N* = 408

Variables	Univariate model
	IRR (95% CI)
Type of VAD	
Port (reference)	
PICC	1.3 (0.36–4.7)
TCC	1.9 (0.14–27.2)
Gender	
Female (reference)	
Male	1.1 (0.33–3.9)
SGA at HPN Start	
Well-nourished (reference)	
Moderately malnourished	0.94 (0.22–4.1)
Severely malnourished	2.3 (0.53–10.1)
ECOG performance status	
0–2 (reference)	
3–5	1.04 (0.25–4.3)
Stage at diagnosis	
Non-metastatic (reference)	
Metastatic	2.0 (0.59–6.7)
Age at HPN Start (years)	0.99 (0.93–1.1)
BMI at HPN Start (kg/m^2^)	1.005 (0.9–1.1)
Albumin at HPN start (g/dl)	
> =3.5 g/dl (reference)	
< 3.5 g/dl	2.7 (0.47–15.5)

*BMI* body mass index, *CI* confidence interval, *CRBSI* catheter-related bloodstream infection, *ECOG* Eastern Cooperative Oncology Group, *g/dl* grams per deciliter, *HPN* home parenteral nutrition, *IRR* incidence rate ratio, *kg/m* ^*2*^ kilograms per meter squared, *PICC* peripherally inserted central catheter, *SGA* Subjective Global Assessment, *TCC* tunneled central catheter, *VAD* venous access device

## Discussion

Little US comparative data exists on the incidence of CRBSIs in HPN patients with a cancer diagnosis who are actively undergoing oncologic treatments. In this study, we evaluated the incidence of CRBSI and the factors associated with it in cancer patients undergoing HPN at a tertiary care cancer center in the US.

We found an overall CRBSI incidence of 0.54 per 1000 HPN days following a standardized catheter care protocol. Several studies have evaluated the incidence of CRBSI in cancer patients receiving HPN. A prospective study by Cotogni et al. evaluated 269 PICCs in 250 cancer patients for a total of 55,293 catheter days, and found a CRBSI rate of 0.05 per 1000 catheter days. Seventy-one percent patients received chemotherapy during the study period [18]. Another prospective study by the same research group, found a CRBSI rate of 0.35 per 1000 catheter days and 0.60 per 1000 HPN days [23]. Other studies have reported CRBSI rates ranging from 0.41 to 1.7 per 1000 catheter days [21, 22, 24, 25, 30]. We did not find any significant difference in the incidence of CRBSI across the three types of VAD, though it is to be noted that of a total of 408 VADs in this study, only 11 (2.7%) were TCCs. This finding could also possibly suggest that the incidence of CRBSI in patients receiving HPN might not be a function of the type of VAD, but rather the catheter care protocols used in these patients. Clearly, this finding needs to be investigated further in future studies.

The majority of previous studies were conducted in Europe with one originating in Japan and none in the US. Only two studies, both by Cotogni et al. [18, 23], were conducted exclusively in cancer patients, while all other studies used a heterogeneous patient population with cancer patients being a subset of the entire study sample. Combining patients with different disease types can lead to erroneous conclusions regarding the incidence rate of CRBSI in a specific population of interest. Our study is different from the above studies in 2 important ways. Our study is the first observational study based out of the US using a large sample of cancer patients. We focused exclusively on cancer patients, 73% of whom were undergoing active oncologic treatments. This patient population is at a high-risk of experiencing CRBSI and has not been adequately investigated in the literature. Having said that, our study is very comparable to that by Cotogni et al. [23] in terms of the patient population investigated and the overall incidence rate of CRBSI reported (0.54 per 1000 HPN days in our study versus 0.60 per 1000 HPN days in the Cotogni study).

Unfortunately, HPN in the US is provided by multiple companies with variable level of expertise and experience. While ASPEN has come up with HPN guidelines, they are not mandated by any private or government insurance agencies. Once the insurer has approved HPN, any provider can deliver the services. This explains why there is a lack of good US-based studies reporting the incidence of CRBSI in HPN patients.

We also evaluated several factors for their association with the incidence of CRBSI. The factors investigated were type of VAD, age, gender, SGA, ECOG performance status, stage at diagnosis, BMI and serum albumin. Upon univariate analysis, no factors were found to be statistically significantly associated with the incidence of CRBSI.

Several risk factors for CRBSI have been reported in the literature. These factors either relate to the patient (type of underlying disease), or the type of VAD (catheter caliber, number of lumens), or the PN therapy (number of HPN days per week), or education (good training given to the patient and caregiver) or follow-up (involvement of a home care nurse) [17]. However, no studies in the literature have investigated the association between baseline nutritional parameters and the risk of CRBSI. Serum albumin is a commonly used parameter to assess nutritional status in cancer and has been described as an independent prognosticator of survival in lung, pancreatic, gastric, colorectal, and breast cancers [31]. In the hospital setting, higher levels of serum albumin have also been found to be correlated with lower in-hospital mortality, lower length of stay (LOS), higher quality of life, and lower rates of nosocomial infection [32, 33]. Although our study did not find a statistically significant association between serum albumin and the incidence of CRBSI, future studies should attempt to further explore this finding using larger sample sizes.

CRBSI is one of the most frequently occurring, extremely costly to treat and potentially life-threatening complications associated with the use of VADs. The risk of CRBSI is even higher in oncology patients who are actively receiving oncologic therapies due to their immunocompromised status. As a result, developing strategies that would prevent or minimize the incidence of CRBSIs has been a continual challenge for healthcare providers. Working in collaboration with a home infusion company that can help implement standardized catheter care protocol can potentially reduce the CRBSI incidence in such patients. These data can serve as a benchmark for potential use by other hospitals in preventing CRBSIs in these patients.

There are some limitations of this study which require careful acknowledgment. This was a retrospective observational study using data not primarily collected for research purposes. As a result, we did not have data available on all potential confounding variables (such as patient and caregiver training, involvement of a home care nurse, assessment of adherence to the catheter care protocol, patient educational status, patient smoking status, steroid use during treatment, liver function tests, and white blood cell counts) that could have impacted the incidence rate of CRBSI. Moreover, all VADs in our study were not specifically placed for the purpose of HPN. In some patients, VADs were initially placed for the purpose of delivering chemotherapy. However, we did not have data on the duration (or number of days) of VAD which can also potentially affect the occurrence of CRBSI. Our study population consisted of heterogeneous non-hospitalized patients with solid tumors from a single institution. As a result, the findings might not be generalizable to all cancer patients, especially those with hematological malignancies and those receiving HPN in a hospital setting. Finally, the humanistic and economic aspects related to the use of VADs were not investigated.

There are some strengths and unique features of our study. By using a consecutive case series of all eligible patients seen at our institution during a fixed time period, we minimized the possibility of selection bias in our study. Most of our patients were receiving active anticancer treatments while undergoing HPN. All VADs were inserted using the same evidence-based protocol and all patients received the same standardized catheter care protocol at home. Our study had a long follow-up period and no patients were lost to follow-up. To the best of our knowledge, this is the first and the largest US-based study reporting the incidence of and factors associated with CRBSI in a consecutive case series of non-hospitalized cancer patients undergoing HPN.

## Conclusions

We found a low incidence rate of CRBSI following a standardized catheter maintenance protocol in a high-risk oncology population undergoing HPN.

## Abbreviations

BMI: Body mass index
CI: Confidence interval
CRBSI: Catheter-related bloodstream infection
ECOG: Eastern Cooperative Oncology Group
g/dl: grams per deciliter
HPN: Home parenteral nutrition
IDSA: Infectious Diseases Society of America
IRB: Institutional Review Board
IRR: Incidence rate ratio
kg/m^2^: kilograms per meter squared
PICC: Peripherally inserted central catheter
SD: Standard deviation
SGA: Subjective Global Assessment
TCC: Tunneled central catheter
VAD: Venous access device
